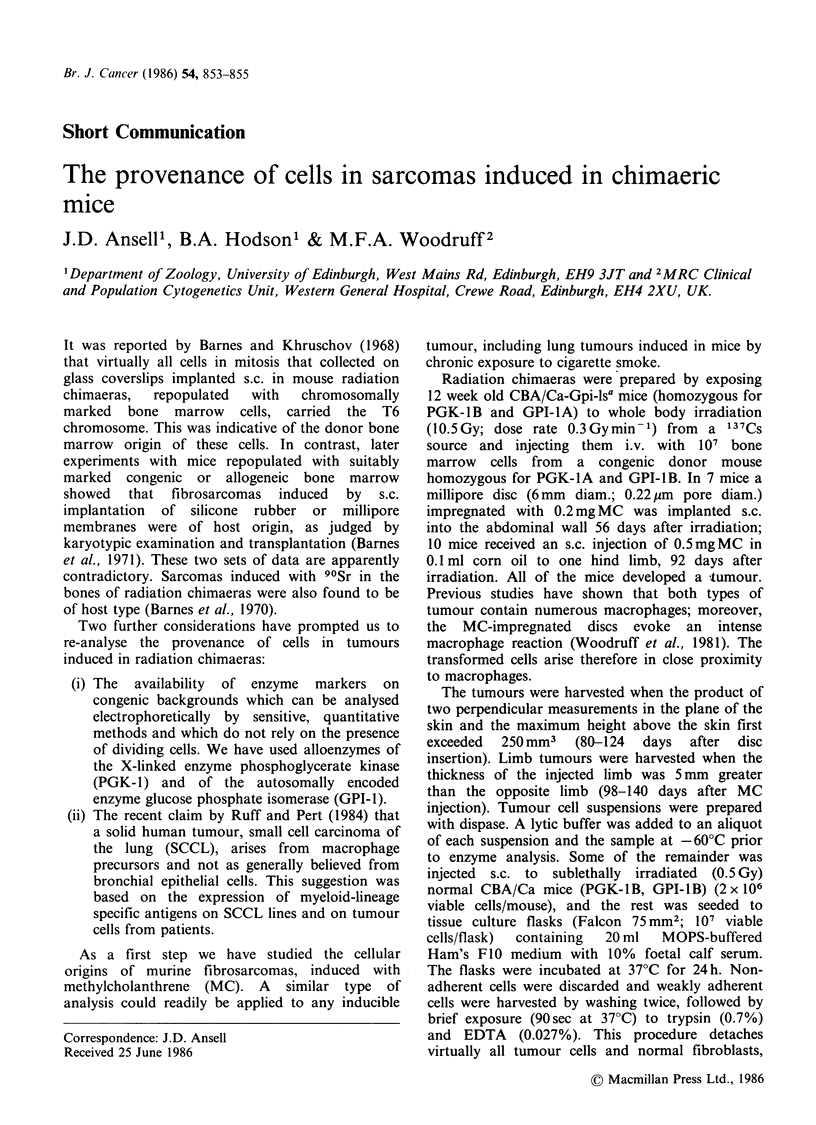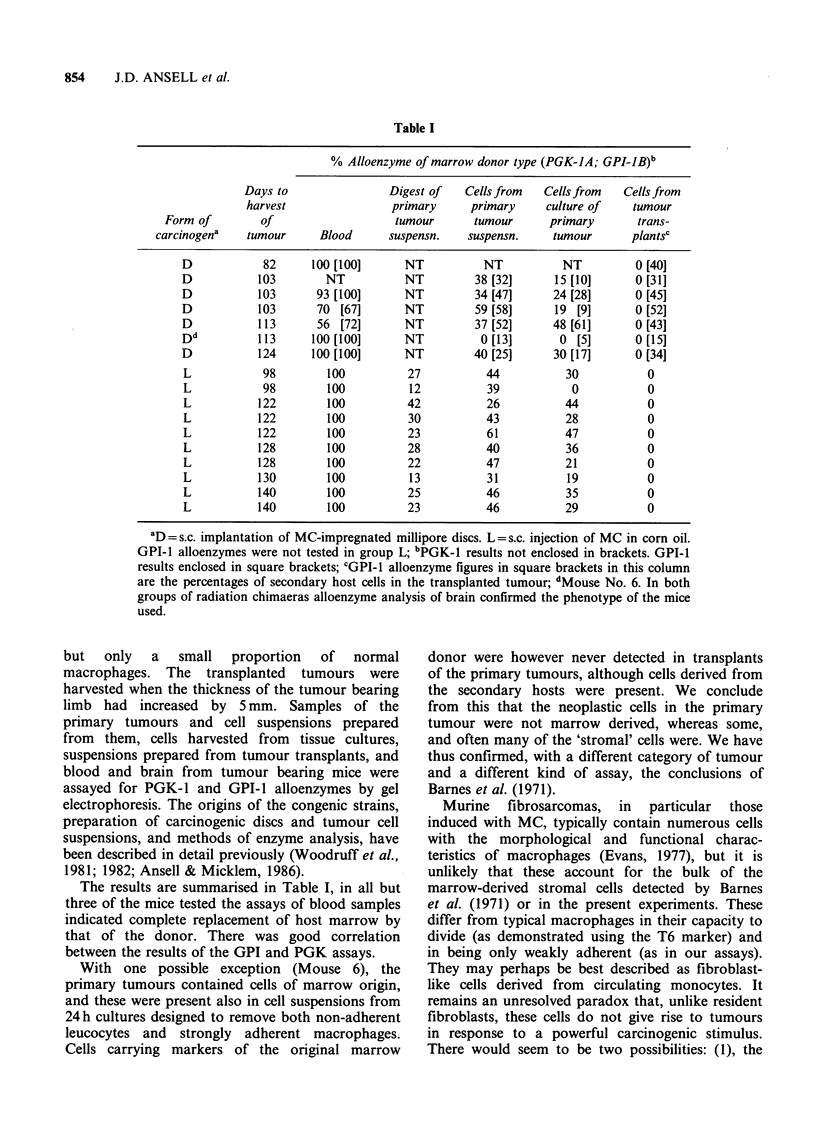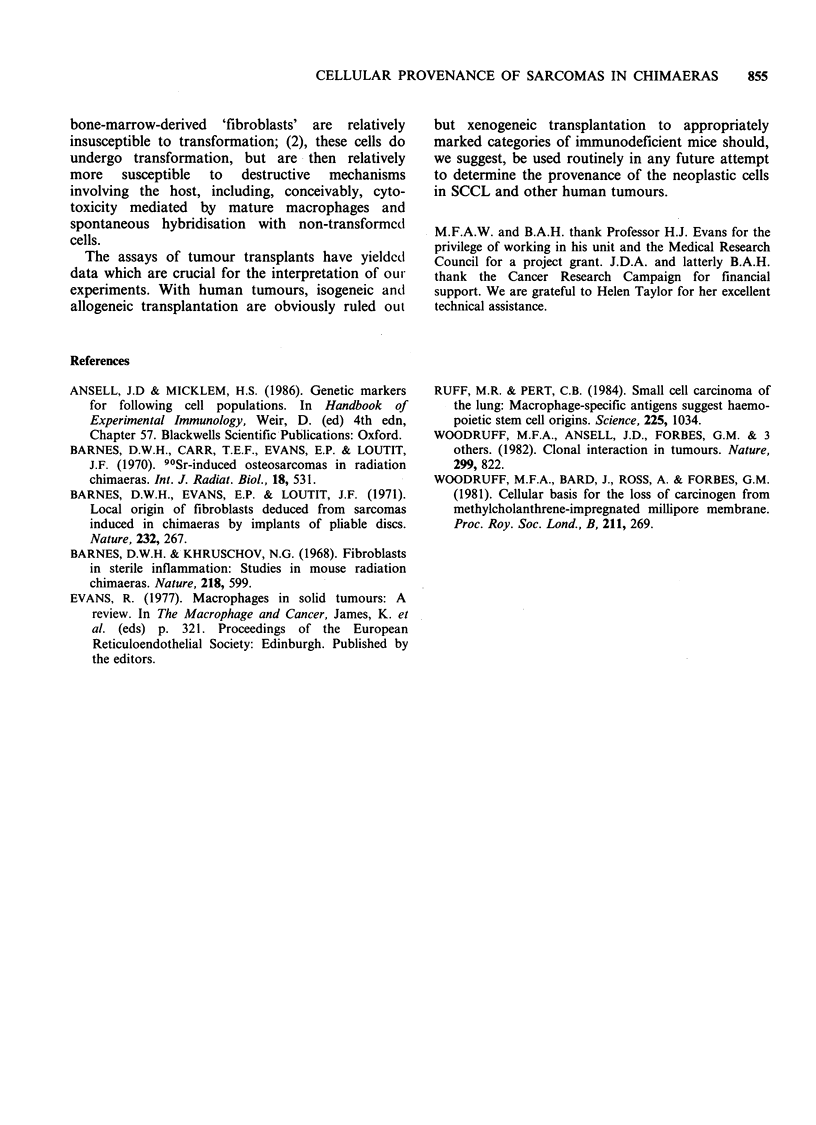# The provenance of cells in sarcomas induced in chimaeric mice.

**DOI:** 10.1038/bjc.1986.251

**Published:** 1986-11

**Authors:** J. D. Ansell, B. A. Hodson, M. F. Woodruff


*Br. J. Cancer* (1986) **54**, 853–855

### Short Communication

### The provenance of cells in sarcomas induced in chimaeric mice

J.D. Ansell[1], B.A. Hodson[1] & M.F.A. Woodruff[2]

[1]*Department of Zoology, University of Edinburgh, West Mains Rd, Edinburgh, EH9 3JT and* [2]*MRC Clinical and Population Cytogenetics Unit, Western General Hospital, Crewe Road, Edinburgh, EH4 2XU, UK.*

It was reported by Barnes and Khruschov (1968) that virtually all cells in mitosis that collected on glass coverslips implanted s.c. in mouse radiation chimaeras, repopulated with chromosomally marked bone marrow cells, carried the T6 chromosome. This was indicative of the donor bone marrow origin of these cells. In contrast, later experiments with mice repopulated with suitably marked congenic or allogeneic bone marrow showed that fibrosarcomas induced by s.c. implantation of silicone rubber or millipore membranes were of host origin, as judged by karyotypic examination and transplantation (Barnes *et al.*, 1971). These two sets of data are apparently contradictory. Sarcomas induced with $^{90}Sr$ in the bones of radiation chimaeras were also found to be of host type (Barnes *et al.*, 1970).

Two further considerations have prompted us to re-analyse the provenance of cells in tumours induced in radiation chimaeras:

(i) The availability of enzyme markers on congenic backgrounds which can be analysed electrophoretically by sensitive, quantitative methods and which do not rely on the presence of dividing cells. We have used alloenzymes of the X-linked enzyme phosphoglycerate kinase (PGK-1) and of the autosomally encoded enzyme glucose phosphate isomerase (GPI-1).

(ii) The recent claim by Ruff and Pert (1984) that a solid human tumour, small cell carcinoma of the lung (SCCL), arises from macrophage precursors and not as generally believed from bronchial epithelial cells. This suggestion was based on the expression of myeloid-lineage specific antigens on SCCL lines and on tumour cells from patients.

As a first step we have studied the cellular origins of murine fibrosarcomas, induced with methylcholanthrene (MC). A similar type of analysis could readily be applied to any inducible tumour, including lung tumours induced in mice by chronic exposure to cigarette smoke.

Radiation chimaeras were prepared by exposing 12 week old CBA/Ca-Gpi-ls$^a$ mice (homozygous for PGK-1B and GPI-1A) to whole body irradiation (10.5 Gy; dose rate 0.3 Gy min$^{-1}$) from a $^{137}Cs$ source and injecting them i.v. with $10^7$ bone marrow cells from a congenic donor mouse homozygous for PGK-1A and GPI-1B. In 7 mice a millipore disc (6 mm diam.; 0.22 $\mu$m pore diam.) impregnated with 0.2 mg MC was implanted s.c. into the abdominal wall 56 days after irradiation; 10 mice received an s.c. injection of 0.5 mg MC in 0.1 ml corn oil to one hind limb, 92 days after irradiation. All of the mice developed a tumour. Previous studies have shown that both types of tumour contain numerous macrophages; moreover, the MC-impregnated discs evoke an intense macrophage reaction (Woodruff *et al.*, 1981). The transformed cells arise therefore in close proximity to macrophages.

The tumours were harvested when the product of two perpendicular measurements in the plane of the skin and the maximum height above the skin first exceeded 250 mm$^3$ (80–124 days after disc insertion). Limb tumours were harvested when the thickness of the injected limb was 5 mm greater than the opposite limb (98–140 days after MC injection). Tumour cell suspensions were prepared with dispase. A lytic buffer was added to an aliquot of each suspension and the sample at −60°C prior to enzyme analysis. Some of the remainder was injected s.c. to sublethally irradiated (0.5 Gy) normal CBA/Ca mice (PGK-1B, GPI-1B) ($2 \times 10^6$ viable cells/mouse), and the rest was seeded to tissue culture flasks (Falcon 75 mm$^2$; $10^7$ viable cells/flask) containing 20 ml MOPS-buffered Ham's F10 medium with 10% foetal calf serum. The flasks were incubated at 37°C for 24 h. Non-adherent cells were discarded and weakly adherent cells were harvested by washing twice, followed by brief exposure (90 sec at 37°C) to trypsin (0.7%) and EDTA (0.027%). This procedure detaches virtually all tumour cells and normal fibroblasts,

Correspondence: J.D. Ansell
Received 25 June 1986

© Macmillan Press Ltd., 1986

**Table I**

| Form of carcinogen[a] | Days to harvest of tumour | % Alloenzyme of marrow donor type (PGK-1A; GPI-1B)[b] Blood | Digest of primary tumour suspensn. | Cells from primary tumour suspensn. | Cells from culture of primary tumour | Cells from tumour transplants[c] |
|---|---|---|---|---|---|---|
| D | 82 | 100 [100] | NT | NT | NT | 0 [40] |
| D | 103 | NT | NT | 38 [32] | 15 [10] | 0 [31] |
| D | 103 | 93 [100] | NT | 34 [47] | 24 [28] | 0 [45] |
| D | 103 | 70 [67] | NT | 59 [58] | 19 [9] | 0 [52] |
| D | 113 | 56 [72] | NT | 37 [52] | 48 [61] | 0 [43] |
| D[d] | 113 | 100 [100] | NT | 0 [13] | 0 [5] | 0 [15] |
| D | 124 | 100 [100] | NT | 40 [25] | 30 [17] | 0 [34] |
| L | 98 | 100 | 27 | 44 | 30 | 0 |
| L | 98 | 100 | 12 | 39 | 0 | 0 |
| L | 122 | 100 | 42 | 26 | 44 | 0 |
| L | 122 | 100 | 30 | 43 | 28 | 0 |
| L | 122 | 100 | 23 | 61 | 47 | 0 |
| L | 128 | 100 | 28 | 40 | 36 | 0 |
| L | 128 | 100 | 22 | 47 | 21 | 0 |
| L | 130 | 100 | 13 | 31 | 19 | 0 |
| L | 140 | 100 | 25 | 46 | 35 | 0 |
| L | 140 | 100 | 23 | 46 | 29 | 0 |

[a]D = s.c. implantation of MC-impregnated millipore discs. L = s.c. injection of MC in corn oil. GPI-1 alloenzymes were not tested in group L; [b]PGK-1 results not enclosed in brackets. GPI-1 results enclosed in square brackets; [c]GPI-1 alloenzyme figures in square brackets in this column are the percentages of secondary host cells in the transplanted tumour; [d]Mouse No. 6. In both groups of radiation chimaeras alloenzyme analysis of brain confirmed the phenotype of the mice used.

but only a small proportion of normal macrophages. The transplanted tumours were harvested when the thickness of the tumour bearing limb had increased by 5 mm. Samples of the primary tumours and cell suspensions prepared from them, cells harvested from tissue cultures, suspensions prepared from tumour transplants, and blood and brain from tumour bearing mice were assayed for PGK-1 and GPI-1 alloenzymes by gel electrophoresis. The origins of the congenic strains, preparation of carcinogenic discs and tumour cell suspensions, and methods of enzyme analysis, have been described in detail previously (Woodruff *et al.*, 1981; 1982; Ansell & Micklem, 1986).

The results are summarised in Table I, in all but three of the mice tested the assays of blood samples indicated complete replacement of host marrow by that of the donor. There was good correlation between the results of the GPI and PGK assays.

With one possible exception (Mouse 6), the primary tumours contained cells of marrow origin, and these were present also in cell suspensions from 24 h cultures designed to remove both non-adherent leucocytes and strongly adherent macrophages. Cells carrying markers of the original marrow donor were however never detected in transplants of the primary tumours, although cells derived from the secondary hosts were present. We conclude from this that the neoplastic cells in the primary tumour were not marrow derived, whereas some, and often many of the 'stromal' cells were. We have thus confirmed, with a different category of tumour and a different kind of assay, the conclusions of Barnes *et al.* (1971).

Murine fibrosarcomas, in particular those induced with MC, typically contain numerous cells with the morphological and functional characteristics of macrophages (Evans, 1977), but it is unlikely that these account for the bulk of the marrow-derived stromal cells detected by Barnes *et al.* (1971) or in the present experiments. These differ from typical macrophages in their capacity to divide (as demonstrated using the T6 marker) and in being only weakly adherent (as in our assays). They may perhaps be best described as fibroblast-like cells derived from circulating monocytes. It remains an unresolved paradox that, unlike resident fibroblasts, these cells do not give rise to tumours in response to a powerful carcinogenic stimulus. There would seem to be two possibilities: (1), the bone-marrow-derived 'fibroblasts' are relatively insusceptible to transformation; (2), these cells do undergo transformation, but are then relatively more susceptible to destructive mechanisms involving the host, including, conceivably, cytotoxicity mediated by mature macrophages and spontaneous hybridisation with non-transformed cells.

The assays of tumour transplants have yielded data which are crucial for the interpretation of our experiments. With human tumours, isogeneic and allogeneic transplantation are obviously ruled out but xenogeneic transplantation to appropriately marked categories of immunodeficient mice should, we suggest, be used routinely in any future attempt to determine the provenance of the neoplastic cells in SCCL and other human tumours.

M.F.A.W. and B.A.H. thank Professor H.J. Evans for the privilege of working in his unit and the Medical Research Council for a project grant. J.D.A. and latterly B.A.H. thank the Cancer Research Campaign for financial support. We are grateful to Helen Taylor for her excellent technical assistance.

**References**

ANSELL, J.D & MICKLEM, H.S. (1986). Genetic markers for following cell populations. In *Handbook of Experimental Immunology,* Weir, D. (ed) 4th edn, Chapter 57. Blackwells Scientific Publications: Oxford.

BARNES, D.W.H., CARR, T.E.F., EVANS, E.P. & LOUTIT, J.F. (1970). $^{90}$Sr-induced osteosarcomas in radiation chimaeras. *Int. J. Radiat. Biol.,* **18,** 531.

BARNES, D.W.H., EVANS, E.P. & LOUTIT, J.F. (1971). Local origin of fibroblasts deduced from sarcomas induced in chimaeras by implants of pliable discs. *Nature,* **232,** 267.

BARNES, D.W.H. & KHRUSCHOV, N.G. (1968). Fibroblasts in sterile inflammation: Studies in mouse radiation chimaeras. *Nature,* **218,** 599.

EVANS, R. (1977). Macrophages in solid tumours: A review. In *The Macrophage and Cancer,* James, K. *et al.* (eds) p. 321. Proceedings of the European Reticuloendothelial Society: Edinburgh. Published by the editors.

RUFF, M.R. & PERT, C.B. (1984). Small cell carcinoma of the lung: Macrophage-specific antigens suggest haemopoietic stem cell origins. *Science,* **225,** 1034.

WOODRUFF, M.F.A., ANSELL, J.D., FORBES, G.M. & 3 others. (1982). Clonal interaction in tumours. *Nature,* **299,** 822.

WOODRUFF, M.F.A., BARD, J., ROSS, A. & FORBES, G.M. (1981). Cellular basis for the loss of carcinogen from methylcholanthrene-impregnated millipore membrane. *Proc. Roy. Soc. Lond., B,* **211,** 269.